# The “Double Hit” in leadless pacing: Acute heart failure from mechanical sensing failure and ventricular dyssynchrony rescued by cardiac resynchronization therapy

**DOI:** 10.1016/j.hrcr.2026.01.007

**Published:** 2026-01-21

**Authors:** Carlos Andres Mejía-Gomez, Camila Pérez-Tellez, María Juliana Reyes-Cardona, María Camila Bejarano-Oliveros, Luis Fernando Pava-Molano, Carlos Enrique Vesga-Reyes

**Affiliations:** 1 Facultad de Ciencias de la Salud, Universidad Icesi, Cali, Colombia; 2 Departamento de Medicina Interna; 3 Departamento de Cardiología; 4 Centro de Investigaciones Clínicas; 5 Unidad de Electrofisiología, Fundación Valle del Lili, Cali, Colombia

**Keywords:** Cardiac pacing, Artificial cardiac resynchronization therapy devices, Artificial pacemaker, Atrioventricular dissociation, Left ventricular dysfunction, Atrioventricular block

**Key Teaching Points**

▪ Leadless transcatheter pacemakers reduce complications but are not free from limitations. Although leadless systems reduce lead- and pocket-related complications, including infections, obligate right ventricular pacing can still cause electromechanical dyssynchrony and precipitate early pacing-induced cardiomyopathy, particularly in pacemaker-dependent patients.
▪ Pacemaker syndrome and pacing-induced cardiomyopathy may coexist and act synergistically. Loss of ventricular synchrony owing to mechanical atrial sensing failure can worsen hemodynamics, whereas concurrent ventricular dyssynchrony may rapidly lead to left ventricular systolic dysfunction in an already vulnerable myocardial substrate.
▪ Individualized and multidisciplinary approaches are necessary for the correct therapy choice in different patient populations. Identification of preimplant markers of myocardial vulnerability, such as a wide native QRS or reduced global longitudinal strain, should prompt a careful selection of pacing strategy, given that right-ventricular-only pacing may overwhelm the already reduced physiological reserves, leading to adverse outcomes such as rapid heart failure.
▪ Cardiac resynchronization therapy is an effective rescue strategy in pacing-induced heart failure. The restoration of mechanical synchrony and reversal of ventricular dysfunction lead to symptom resolution, even when heart failure develops shortly after pacemaker implantation.

## Introduction

Cardiac implantable electronic devices are essential for managing high-grade conduction disorders.^1^ Although transvenous pacing remains the standard, leadless pacemakers such as the Micra transcatheter pacing system (Medtronic) have transformed therapy by eliminating surgical pockets and transvenous leads, thereby reducing infection and lead-related complications, particularly valuable in patients with compromised venous access or high infection risk.^2^

Despite these structural advantages, the hemodynamic limitations of leadless pacing require careful consideration. The Micra AV uses an accelerometer to detect mechanical atrial activity, aiming for physiological atrioventricular (AV) synchrony (VDD pacing).^3^ However, reliance on mechanical rather than electrical sensing renders the system susceptible to tracking failures.^4^^,^^5^ Loss of AV synchrony can precipitate pacemaker syndrome, a well-described complication of single-chamber ventricular pacing.^6^^,^^7^

In addition, obligate right ventricular (RV) pacing inherent to current leadless systems introduces electromechanical dyssynchrony, a recognized driver of pacing-induced cardiomyopathy (PICM).^8^ Although PICM is classically described as a chronic remodeling process, a subset of patients may experience accelerated hemodynamic deterioration.^9^ In pacing-dependent individuals, the synergistic burden of ventricular dyssynchrony and lost AV coordination may precipitate heart failure within weeks of implantation.^10^

We present the case of a 69-year-old woman who developed severe congestion, persistent AV dissociation, and new-onset left ventricular (LV) systolic dysfunction (LV ejection fraction [LVEF] 44%), 1 month after Micra AV implantation.

## Case report

The patient had a history of breast cancer treated with surgery and radiotherapy in 2003, currently in remission, and was diagnosed as having high-grade AV block. A Micra AV leadless transcatheter pacemaker was implanted at an outside institution owing to active cellulitis over the left infraclavicular region, precluding standard transvenous access.

**Figure 1:**
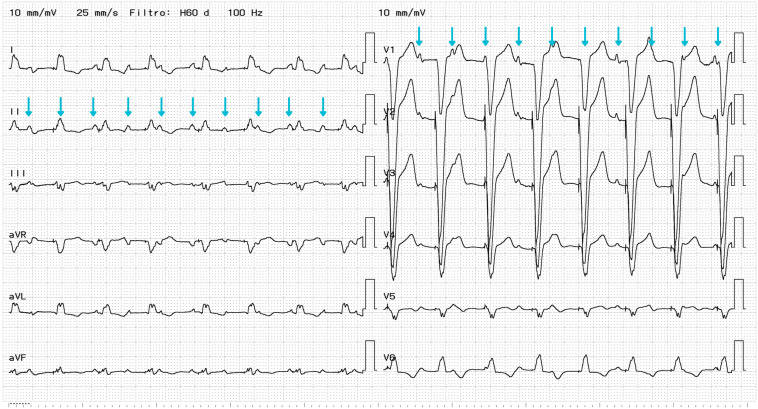
12-lead electrocardiogram: atrioventricular dissociation, right ventricular pacing (spikes preceding each QRS complex), and wide QRS (160 ms). *Blue arrows* point to sensed P waves.

1 month later, she presented with a 15-day history of worsening dyspnea, orthopnea, and significant lower extremity edema. On admission, blood pressure was 135/56 mm Hg, heart rate 95 beats/min (bpm), respiratory rate 28 breaths/min, oxygen saturation 94%, and temperature 35.8°C. Physical examination showed jugular venous distension with cannon A waves, S3 gallop, and bilateral grade 2 pitting edema. Admission N-terminal pro-B-type natriuretic peptide was markedly elevated at 9531 pg/mL. Although renal function, electrolytes, and thyroid profile were normal. Chest radiography revealed bilateral pleural effusions, and the 12-lead electrocardiogram revealed AV dissociation (Figure 1).

**Figure 2:**
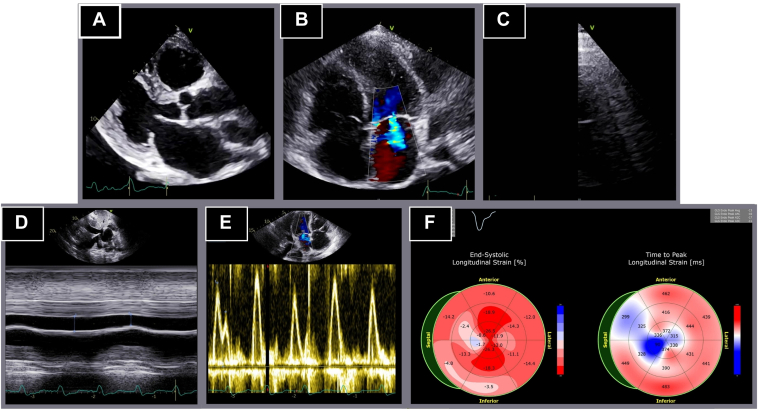
Transthoracic echocardiogram. Left ventricular ejection fraction of 44%. **A:** Parasternal long axis view. **B:** Apical 4-chamber view. **C:** B lines on pleural ultrasound. **D:** Severely dilated inferior vena cava (26 mm, with an inspiratory collapse 21%). **E:** Atrioventricular dissociation in transmitral inflow. **F:** Altered longitudinal strain of −11.1%.

Transthoracic echocardiography revealed new-onset LV systolic dysfunction with an LVEF of 44%, an indexed LV end-diastolic volume of 36.2 mL/m^2^, and a reduced global longitudinal strain (GLS) of −11.1%. Significant findings included paradoxical interventricular septal motion, a moderately dilated left atrium, and Doppler evidence of AV dissociation. Signs of systemic congestion included B lines on lung ultrasound, a severely dilated inferior vena cava without respiratory collapse, and a small pericardial effusion (Figure 2).

Electrophysiological evaluation confirmed AV dissociation with an intrinsic atrial rate of 120 bpm and a ventricular paced rate of 78 bpm. Device interrogation showed failure of mechanical atrial sensing; despite aggressive reprogramming, atrial tracking could not be restored. The patient was diagnosed as having pacemaker syndrome complicated by pacing-induced ventricular dysfunction. She was admitted to the cardiovascular intensive care unit for diuretic therapy, which yielded symptomatic improvement.

**Figure 3:**
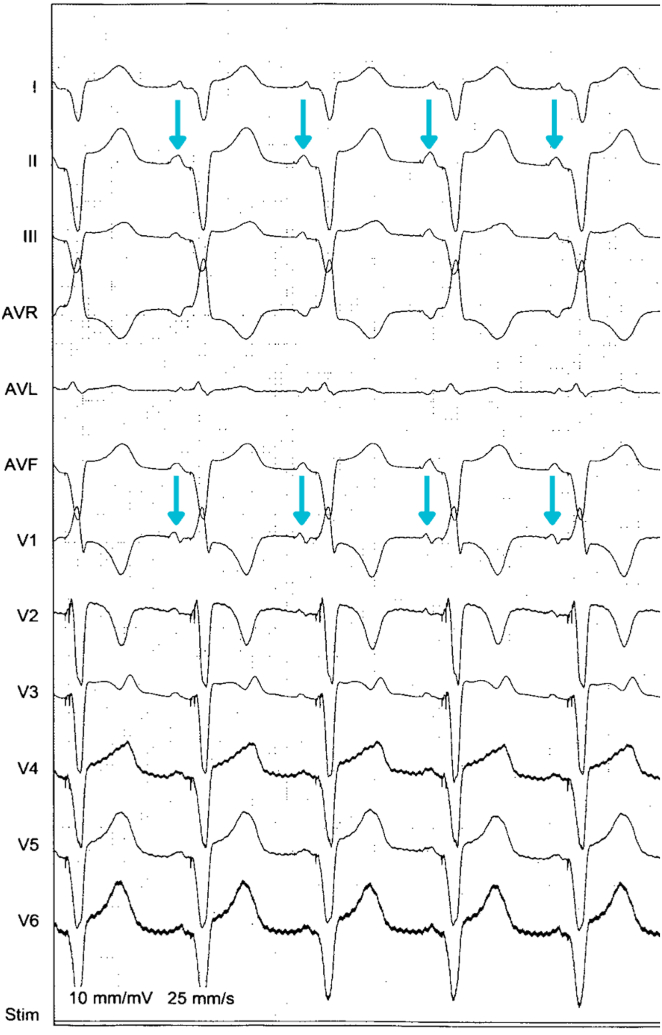
12-lead electrocardiogram after cardiac resynchronization therapy pacemaker showing an atrial-sensed, biventricular-paced rhythm. Small ventricular pacing spikes are visible immediately before each narrow QRS, and P waves precede every paced complex (*blue arrows*).

Given the need for both reliable AV synchrony and physiological ventricular activation, a multidisciplinary decision was made to upgrade to a cardiac resynchronization therapy (CRT) pacemaker. A Medtronic CRT pacemaker system was implanted via the right subclavian vein; with leads placed in the right atrium, RV, and a tributary of the coronary sinus. The device was programmed to DDDR mode (70–120 bpm) with an LV-RV offset of 30 ms. Postprocedural electrocardiogram showed sinus rhythm with consistent atrial sensing and biventricular capture (Figure 3).

**Figure 4:**
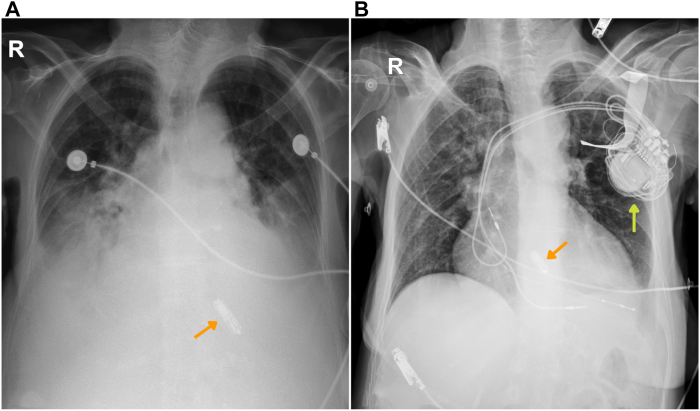
**A:** Chest radiograph on arrival, bilateral pleural effusion and pulmonary edema. **B:** Postprocedural chest radiograph showing a cardiac resynchronization therapy pacemaker device. *Orange arrows* = Micra AV device; *yellow arrow* = cardiac resynchronization therapy pacemaker device.

**Figure 5:**
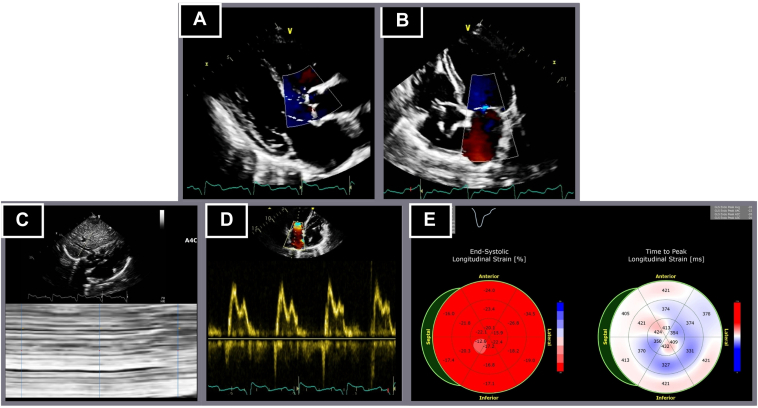
Control transthoracic echocardiogram. Left ventricular ejection fraction of 55%. **A:** Parasternal long axis view. **B:** Apical 4-chamber view. **C:** Collapsed inferior vena cava. **D:** Transmitral flow Doppler shows peak E and peak A waves previously absent. **E:** Normalized longitudinal strain of −20.1%.

The postprocedural course was uncomplicated, with resolution of edema and improvement to New York Heart Association class I. Discharge weight was 40.2 kg, reflecting approximately 15 kg of net fluid loss. Follow-up chest radiograph confirmed appropriate lead position and resolution of congestion (Figure 4). Repeat echocardiography 1 month later demonstrated normalization of LVEF to 55%. Transmitral Doppler showed distinct E and A waves, confirming restoration of AV synchrony and no residual signs of congestion, with a nondilated inferior vena cava and absence of B lines (Figure 5). The patient remains asymptomatic after 2 years of outpatient follow-up.

## Discussion

This case highlights the complex interplay between mechanical sensing limitations and myocardial substrate vulnerability in leadless pacing. Although Micra AV offers significant structural advantages over transvenous systems,^11^^,^^12^ our findings underscore that, in patients with preexisting conduction disease, the combination of AV dissociation and ventricular dyssynchrony can precipitate a rapid and severe form of pacing-induced heart failure.

Pacemaker syndrome results from the loss of AV synchrony, a known complication of single-chamber pacing, depriving the LV of the atrial “kick.” In the Mode Selection Trial, pacemaker syndrome affects nearly 18% of VVI-paced patients.^13^ In this patient, who likely had underlying diastolic stiffness owing to previous thoracic radiation, the abrupt loss of atrial contribution was hemodynamically catastrophic. Although accelerometer-based algorithms achieved high (up to 95%) synchrony in the MARVEL 2 trial,^3^ real-world performance can degrade during tachycardia or low-amplitude mechanical atrial activity, as observed here.

The second driver of deterioration was the acute onset of ventricular systolic dysfunction. Although PICM is typically described as a chronic remodeling process occurring in 10%–20% of RV-paced patients over the years, “rapid decliners” exist. Retrospectively, this patient exhibited high-risk markers for PICM before implantation: a wide native QRS and reduced GLS despite a preserved LVEF.^14^ These markers highlight an underlying vulnerable substrate and were likely unavailable to the implanting team at the outside institution, underscoring the clinical risks posed by fragmented health records and the vital importance of interinstitutional data sharing for optimal risk stratification.

Although the Micra was positioned in the RV outflow tract, a site associated with narrower paced QRS durations (mean 142 ms) than the apex,^15^ it was insufficient to prevent dyssynchrony. The high burden of ventricular pacing (>90%) overwhelmed the limited physiological reserve of this compromised substrate, precipitating acute failure.

The decision to pursue CRT was driven by the imperative to reverse pacing-induced mechanical dysfunction. Although conduction system pacing, specifically His-bundle or left bundle branch area pacing, has emerged as a strictly physiological alternative that recruits the native conduction system to preserve narrow QRS duration, CRT remains the guideline-supported standard for rescuing patients with established ventricular dysfunction.^16^^,^^17^

Recent evidence suggests that both left bundle branch area pacing and biventricular pacing can effectively reverse PICM, but, in this case, biventricular pacing provided a robust and successful correction of the dyssynchrony. The recovery of LVEF to 55% and resolution of symptoms reinforce that the failure was mechanistic (electrical) rather than intrinsic. For patients with wide native QRS or reduced GLS requiring high-burden pacing, strategies that preserve physiological activation, via conduction system pacing or CRT, should be prioritized when anatomy and infection risk permit, avoiding the predictable desynchronization of RV-only pacing.^18^

## Conclusion

Leadless AV pacemakers are a valuable tool but are not devoid of hemodynamic risks. In patients with vulnerable myocardial substrates (wide native QRS, reduced GLS), the “double hit” of mechanical AV dissociation and RV dyssynchrony can precipitate rapid heart failure. Clinicians must recognize that “safety” extends beyond the absence of infection to include hemodynamic stability. In such “rapid decliners,” upgrading to CRT is a definitive strategy to restore synchrony and recover ventricular function.

## Disclosures

The authors have no conflicts of interest to disclose.

## References

[1] Al-Khatib S.M., Leopold J.A. (2024). Cardiac implantable electronic devices. N Engl J Med.

[2] Tjong F.V.Y., Reddy V.Y. (2017). Permanent leadless cardiac pacemaker therapy: A comprehensive review. Circulation.

[3] Steinwender C., Khelae S.K., Garweg C. (2020). Atrioventricular synchronous pacing using a leadless ventricular pacemaker: results from the MARVEL 2 study. JACC Clin Electrophysiol.

[4] Van Weyenbergh T., Willems R., Vandenberk B., Dorrestijn A., Vörös G., Garweg C. (2025). Atrial fibrillation and Micra AV leadless pacemaker: A challenge for atrioventricular synchrony?. Heart Rhythm.

[5] Sudo Y. (2025). Position-dependent and hidden atrioventricular dyssynchrony in Micra AV leadless pacemaker. PACE Pacing Clin Electrophysiol.

[6] Mitacchione G., Schiavone M., Gasperetti A. (2021). Micra-AV leadless pacemaker and atrioventricular (dys)synchrony: A stepwise process. PACE Pacing Clin Electrophysiol.

[7] Boersma L.V., El-Chami M., Steinwender C. (2022). Practical considerations, indications, and future perspectives for leadless and extravascular cardiac implantable electronic devices: a position paper by EHRA/HRS/LAHRS/APHRS. Europace.

[8] Pipilas D., Frankel D.S., Khurshid S. (2023). Pacing-induced cardiomyopathy after leadless pacemaker implant: it’s all about location, location, location. J Cardiovasc Electrophysiol.

[9] Ko E., Isotani A., Shirai S., Ando K. (2024). Pacing-induced cardiomyopathy in a patient with a leadless pacemaker following transcatheter aortic valve replacement. Clin Case Rep.

[10] Gavaghan C. (2022). Pacemaker induced cardiomyopathy: an overview of current literature. Curr Cardiol Rev.

[11] Crossley G.H., Longacre C., Higuera L. (2024). Outcomes of patients implanted with an atrioventricular synchronous leadless ventricular pacemaker in the Medicare population. Heart Rhythm.

[12] Knops R.E., Tjong F.V.Y., Neuzil P. (2015). Chronic performance of a leadless cardiac pacemaker. J Am Coll Cardiol.

[13] Link M.S., Hellkamp A.S., Estes N.A.M. (2004). High incidence of pacemaker syndrome in patients with sinus node dysfunction treated with ventricular-based pacing in the Mode Selection Trial (MOST). J Am Coll Cardiol.

[14] Khurshid S., Frankel D.S. (2023). Pacing-induced cardiomyopathy. Cardiol Clin.

[15] Garweg C., Vandenberk B., Foulon S., Haemers P., Ector J., Willems R. (2019). Leadless pacing with Micra TPS: A comparison between right ventricular outflow tract, mid-septal, and apical implant sites. J Cardiovasc Electrophysiol.

[16] Glikson M., Burri H., Abdin A. (2025). European Society of Cardiology (ESC) clinical consensus statement on indications for conduction system pacing, with special contribution of the European Heart Rhythm Association of the ESC and endorsed by the Asia Pacific Heart Rhythm Society, the Canadian Heart Rhythm Society, the Heart Rhythm Society, and the Latin American Heart Rhythm Society. Europace.

[17] Christoph M., Marius S., Karl S., Friedrich K. (2023). Efficacy of CRT upgrade in pacemaker-induced cardiomyopathy in an outpatient clinic - Results of a prospective registry. Int J Cardiol.

[18] Lee K., Ikram M., Reddy M. (2025). Pacing-induced cardiomyopathy following leadless and transvenous pacemaker implantation: a multicenter retrospective study. Heart Rhythm O2.

